# Disseminated intravascular coagulation or acute coagulopathy of trauma shock early after trauma? An observational study

**DOI:** 10.1186/cc10553

**Published:** 2011-11-17

**Authors:** Pär I Johansson, Anne Marie Sørensen, Anders Perner, Karen Lise Welling, Michael Wanscher, Claus F Larsen, Sisse R Ostrowski

**Affiliations:** 1 Section for Transfusion Medicine, Capital Region Blood Bank, Copenhagen University Hospital, Rigshospitalet, Blegdamsvej 9, DK-2100 Copenhagen, Denmark; 2 Department of Anesthesia, Copenhagen University Hospital, Rigshospitalet, Blegdamsvej 9, DK-2100 Copenhagen, Denmark; 3 The Trauma Center, Center of Head and Orthopedics, Copenhagen University Hospital, Rigshospitalet, Blegdamsvej 9, DK-2100 Copenhagen, Denmark; 4 Department of Intensive Care, Copenhagen University Hospital, Rigshospitalet, Blegdamsvej 9, DK-2100 Copenhagen, Denmark; 5 Department of Neurointensive Care, Copenhagen University Hospital, Rigshospitalet, Blegdamsvej 9, DK-2100 Copenhagen, Denmark; 6 Department of Cardiothoracic Anesthesia, Copenhagen University Hospital, Rigshospitalet, Blegdamsvej 9, DK-2100 Copenhagen, Denmark

**Keywords:** DIC, ACoTS, TIC, trauma, coagulopathy, glycocalyx, protein C, fibrinolysis, inflammation, consumption

## Abstract

**Introduction:**

It is debated whether early trauma-induced coagulopathy (TIC) in severely injured patients reflects disseminated intravascular coagulation (DIC) with a fibrinolytic phenotype, acute coagulopathy of trauma shock (ACoTS) or yet other entities. This study investigated the prevalence of overt DIC and ACoTS in trauma patients and characterized these conditions based on their biomarker profiles.

**Methods:**

An observational study was carried out at a single Level I Trauma Center. Eighty adult trauma patients (≥18 years) who met criteria for full trauma team activation and had an arterial cannula inserted were included. Blood was sampled a median of 68 minutes (IQR 48 to 88) post-injury. Data on demography, biochemistry, injury severity score (ISS) and mortality were recorded. Plasma/serum was analyzed for biomarkers reflecting tissue/endothelial cell/glycocalyx damage (histone-complexed DNA fragments, Annexin V, thrombomodulin, syndecan-1), coagulation activation/inhibition (prothrombinfragment 1+2, thrombin/antithrombin-complexes, antithrombin, protein C, activated protein C, endothelial protein C receptor, protein S, tissue factor pathway inhibitor, vWF), factor consumption (fibrinogen, FXIII), fibrinolysis (D-dimer, tissue-type plasminogen activator, plasminogen activator inhibitor-1) and inflammation (interleukin (IL)-6, terminal complement complex (sC5b-9)). Comparison of patients stratified according to the presence or absence of overt DIC (International Society of Thrombosis and Hemostasis (ISTH) criteria) or ACoTS (activated partial thromboplastin time (APTT) and/or international normalized ratio (INR) above normal reference).

**Results:**

No patients had overt DIC whereas 15% had ACoTS. ACoTS patients had higher ISS, transfusion requirements and mortality (all *P *< 0.01) and a biomarker profile suggestive of enhanced tissue, endothelial cell and glycocalyx damage and consumption coagulopathy with low protein C, antithrombin, fibrinogen and FXIII levels, hyperfibrinolysis and inflammation (all *P *< 0.05). Importantly, in non-ACoTS patients, apart from APTT/INR, higher ISS correlated with biomarkers of enhanced tissue, endothelial cell and glycocalyx damage, protein C activation, coagulation factor consumption, hyperfibrinolysis and inflammation, that is, resembling that observed in patients with ACoTS.

**Conclusions:**

ACoTS and non-ACoTS may represent a continuum of coagulopathy reflecting a progressive early evolutionarily adapted hemostatic response to the trauma hit and both are parts of TIC whereas DIC does not appear to be part of this early response.

## Introduction

Trauma is a major cause of death and disability worldwide [1], and since hemorrhage accounts for approximately 40% of all trauma fatalities. this is a leading cause of potentially preventable deaths [2]. When death is not immediately due to exsanguination, bleeding and prolonged shock increase the risk of multiple organ failure and late mortality [2,3]. The exact mechanism(s) responsible for death in trauma patients remain elusive but much attention has been given to coagulopathy since this is an independent predictor of mortality [4] that is present already at the scene of an accident [5,6] and upon arrival at the emergency department [7,8].

Different drivers of trauma-induced coagulopathy (TIC) have been proposed by different groups of researchers [9]: Some advocate that TIC reflects disseminated intravascular coagulation (DIC) with a fibrinolytic (hemorrhagic) phenotype based on the observation that trauma DIC patients display prolonged prothrombin time (PT), have low fibrinogen and antithrombin (AT) levels early after injury and have high fibrin/fibrinogen degradation products (FDP) and D-dimer levels indicating massive (uncontrolled) thrombin generation and activation (through the tissue-factor dependent coagulation pathway) followed by extensive fibrin(ogen)olysis and consumption coagulopathy [8,10,11]. Furthermore, they infer that the higher FDP/D-dimer ratio and low fibrinogen levels result from fibrin(ogen)olysis caused by excessive plasmin and neutrophil elastase production and release, independent of hypoperfusion [11,12]. Contrary to this view, Brohi and colleagues proposed that TIC, as identified by a moderately prolonged activated partial thromboplastin time (APTT), prothrombin time (PT) or international normalized ratio (INR) [7,13] is driven by the combination of trauma and shock and, importantly, by the degree of tissue hypoperfusion (acute coagulopathy of trauma shock, ACoTS) [14]. The underlying mechanism is suggested to include enhanced early generation of activated factors, including thrombin that, due to reduced thrombin clearance, results in increased thrombin-thrombomodulin complex formation on adjacent endothelial cells and ensuing enhanced activation of the anticoagulant protein C (PC) pathway with enhanced PC activation. This leads to reduced thrombin generation, through inhibition of FV and FVIII, decreased fibrinogen utilization and enhanced fibrinolysis [4,14,15]. Importantly, they emphasize that microvascular thrombosis does not occur in trauma hemorrhage and that there is a relative sparing of platelets and fibrinogen, thereby making ACoTS a distinct clinical entity from the DIC observed in, for example, sepsis and other disease conditions classically associated with DIC [4].

Common for both models presented above, however, is that only the fluid phase of the hemostatic system or, more correctly, only the concentrations of different elements in the plasma fraction of the fluid phase, has been investigated. We recently suggested that the state of the fluid phase including its cellular elements, that is, circulating whole blood, is a consequence of the degree of the tissue injury and, importantly, is critically related to the degree of endothelial damage, with a progressively more procoagulant endothelium inducing a gradient of increasing anticoagulation towards the fluid phase [16]. In alignment with this, we found that in trauma patients upon hospital admission, a high level of syndecan-1, a marker of endothelial glycocalyx degradation, was associated with high sympathoadrenal activity and increased mortality, even after adjusting for injury severity score [17]. Also, only in patients with high syndecan-1 levels was increasing injury severity associated with increased tissue and endothelial damage, protein C depletion, hyperfibrinolysis and inflammation [17].

The aim of the present study was, therefore, to investigate the prevalence of DIC and ACoTS, respectively, in trauma patients upon admission to our Level I Trauma Center and to characterize these conditions based on their biomarker profiles to delineate differences in association between the trauma hit (injury severity, shock) and biomarkers of tissue injury, endothelial and glycocalyx damage, thrombin generation and factor depletion, fibrinolysis and inflammation. Here we report that no trauma patients with blood sampled approximately one hour post-injury had overt DIC, whereas 15% had ACoTS defined as moderately increased APTT and/or INR. Furthermore, ACoTS patients and non-ACoTS patients displayed a comparable biomarker profile with increasing injury severity indicating that these conditions represent a continuum of coagulopathy reflecting a progressive early evolutionarily adapted hemostatic response to the trauma hit and both are parts of TIC, whereas DIC does not appear to be part of this early response.

## Materials and methods

### Study design

This was an observational cohort study of trauma patients admitted directly to a Level I Trauma Center at a tertiary hospital (Rigshospitalet, Copenhagen, Denmark, covering 2.5 million inhabitants) between March 2010 and November 2010. The study is part of an ongoing larger international multicenter study, Activation of Coagulation and Inflammation after Trauma 3 (ACIT3), approved by the Regional Ethics Committee (H-4-2009-139), the Danish Data Protection Agency and conducted in accordance with the Declaration of Helsinki. Written informed consent was obtained from the patients or next of kin. Here we report on the findings related to a cohort of 80 patients recruited from the first 100 enrolled in the ACIT3 study enabling measurements of an extensive number of biomarkers with ELISA kits that each provide analysis of 80 samples.

### Patient selection

ACIT3 study inclusions were as follows: adult trauma patients (≥18 years) who met criteria for full trauma team activation (based on the mechanism of injury or physiologic or anatomic injury criteria) and had an arterial cannula. The latter was chosen since only patients with expected severe injuries have an arterial cannula placed immediately upon hospital admission. Exclusion criteria were arrival in the Trauma Center > 2 hours after injury; > 2, 000 ml of intravenous fluids administered before hospital arrival; transfer from another hospital and burns > 5% total body surface area. Patients were retrospectively excluded if they were taking anticoagulant/antiplatelet medications (except aspirin), had moderate or severe liver disease or had known bleeding diathesis.

The 80 included patients were selected from the first 100 patients recruited to the ACIT3 study with complete data. We intended to include 80 patients because we measured an extensive number of biomarkers by ELISA, with each ELISA kit providing analysis of 80 samples. We aimed at including the most severely injured and/or coagulopathic patients and selected the 80 patients according to: outcome (mortality or intensive care unit (ICU) admission post trauma; yes), transfusion of red blood cells (RBC) within six hours (yes), Revised Trauma Score (RTS) (< 5.00, we had no access to Injury Severity Score (ISS) until later in the study period) or coagulopathy (APTT ≥35 sec, INR ≥1.2, Ly30 > 1%/Cl30 < 95%; yes). This yielded 70 severely injured/coagulopathic patients, and additionally 10 patients (aged 48 years (IQR 43 to 52), 60% males) were selected blinded from the remaining 30 and so on). The 20 patients not included in this study, had, compared to the included patients, comparable age and gender (41 years (IQR 33 to 53), 40% males) and APTT (26 (IQR 23 to 27), NS) but had, as expected, lower ISS (4 (IQR 2 to 10), *P *< 0.001), mortality (0%, *P *= 0.037) and INR (1.1 (IQR 1.0 to 1.1), *P *= 0.007). Two of the 20 patients not included had a hypercoagulable Thrombelastography (TEG) (maximal amplitude (MA) > 69, 10%).

Data on demography, clinical and biochemical parameters, investigations, management and 30-day mortality were recorded and ISS scores were obtained from the Trauma Audit & Research Network (TARN) database.

No patients received tranexamic acid, adrenaline or noradrenaline prior to blood sampling.

### DIC and ACoTS criteria

Overt DIC was defined [18] and modified [19] according to the International Society of Thrombosis and Hemostasis (ISTH) criteria, with the following cut off values: I) Platelet counts < 50 *10^9^/L (two points), 50 to 100 *10^9^/l (one point); II) Fibrinogen < 1 g/l (one point); III) D-dimer > 4 mg/l (three points), 0.39 to 4.00 mg/l (two points) and IV) INR (PT is not available at our hospital) > 2.3 (two points), 1.4 to 2.3 (one point). Overt DIC was diagnosed as a sum of five or more points. ACoTS was diagnosed as APTT and/or INR above normal reference, that is, > 35 sec or > 1.2 ratio, respectively, in accordance with previous studies [17,20,21].

### Blood sampling

Blood was sampled immediately upon arrival for standard arterial blood gas (ABG, Radiometer ABL 725/735, Radiometer AS, Bronshøj,), routine biochemistry and research analyses (citrate, heparin, EDTA plasma, serum). Routine biochemistry samples were analyzed in a DS/EN ISO 15189 standardized laboratory by a Sysmex XE-2100, Sysmex Europe GmbH, Norderstedt, Germany (hemoblobin, platelets, leukocytes) and ACL TOP (APTT, INR, AT, fibrinogen). Plasma samples were ice-cooled immediately whereas serum samples were kept at room temperature (RT) for 1 h before centrifugation (one (serum) or two (plasma) times 1, 800 g at 5°C for 10 minutes) and storage at -80°C.

### Enzyme linked immunosorbent assay (ELISA) measurements

Soluble biomarkers of tissue, endothelial cell and glycocalyx damage, coagulation activation/inhibition and factor consumption, fibrinolysis and inflammation were measured in uniplicate by commercially available immunoassays according to the manufacturer's recommendations. In each patient, all 18 biomarkers were measured corresponding to a total of 18*80 = 1, 440 measurements, with no missing measurements. The biomarkers were analyzed in EDTA/citrate plasma or serum as follows: EDTA plasma: histone-complexed DNA fragments (hcDNA, Cell Death Detection ELISA^PLUS^, Roche, Hvidovre, Denmark; LLD not stated, relative quantification); Annexin V (American Diagnostica Inc. (ADI), Stamford, CT, USA; LLD not stated, normal reference < 10 ng/ml); soluble thrombomodulin (sTM) (Nordic Biosite, Copenhagen, Denmark; LLD 0.38 ng/ml) and D-dimer (ADI; LLD 2-4 ng/ml). Citrate plasma: protein C (PC, Helena Laboratories, Beaumont, TX, USA; LLD 5% of reference plasma); activated protein C (APC, USCNLIFE; LLD 4.2 pg/ml); soluble endothelial protein C receptor (sEPCR, R&D Systems Europe, Abingdon, UK; LLD 0.064 ng/ml); protein S (PS, ADI; LLD not stated, quantified relative to provided reference plasma); tissue-type plasminogen activator (tPA, ADI, detects sc-tPA, tc-tPA and tPA/plasminogen activator inhibitor (PAI)-1 complexes; LLD 1 ng/ml); plasminogen activator inhibitor-1 (PAI-1, Assaypro, St. Charles, MO, USA; LLD 0.2 ng/ml); prothrombinfragment 1 and 2 (PF1.2, USCNLIFE; LLD 0.043 nmol/l); thrombin/antithrombin complex (TAT, USCNLIFE, Wuhan EIAab Science Co, Wuhan, China; LLD 0.215 ng/ml); tissue factor pathway inhibitor (TFPI, ADI, detects intact TFPI, truncated TFPI, TF/FVIIa/TFPI complexes; LLD 0.18 ng/ml); von Willebrand Factor antigen (vWF, Helena Laboratories, LLD 5% of reference plasma); factor XIII (FXIII, Assaypro; LLD 50 pg/ml); terminal complement complex (sC5b-9, MicroVue sC5b-9 plus EIA Kit, Quidel Corp., San Diego, CA, USA; LLD 3.7 ng/ml) and interleukin-6 (IL-6, Quantikine HS, R&D Systems Europe; LLD 0.039 pg/ml). Serum: Syndecan-1 (Diaclone SAS, Besancon, France; LLD 2.56 ng/ml).

### Statistics

Statistical analysis was performed using SAS 9.1 (SAS Institute Inc., Cary, NC, USA). Data from patients stratified according to presence (*n *= 12) or absence (*n *= 68) of ACoTS were compared by Wilcoxon Rank Sum tests and Chi-square/Fischer exact tests, as appropriate and this comparison was performed both on the full cohort of patients and after excluding patients with sTBI. Correlations were investigated by Spearman correlations, presented by rho and *P*-values. Data are presented as medians with interquartile ranges (IQR). *P*-values < 0.05 were considered significant.

## Results

### Study patients

The present study included 80 trauma patients with ISS in the entire range (ISS > 26 *n *= 23, 15 to 26 *n *= 26, and < 15 *n *= 30) and with demography, injury severity, biochemistry, coagulopathy and mortality as displayed in Table 1. Most patients (96%) were referred by mobile emergency care units (MECU) staffed with anesthetists (28% by helicopter) and blood samples were drawn a median of 68 minutes (IQR 48 to 88) after the injury. Eleven patients (14%) received massive transfusion (> 10 RBC the initial 24 hours) and overall 30-day mortality was 18% (*n *= 14) (Table 1).

**Table 1:** Demography, injury severity, biochemistry, hemostasis, transfusion requirements and mortality in the 80 trauma patients investigated

		Patients
**N**		80
**Age**	yrs	46 (33 to 64)
**Gender**	m%	68% (54)
**Blunt trauma**	% (n)	91% (73)
**ISS**	score	17 (10 to 28)
**sTBI**	% (n)	31% (22)
**GCS (PH)**	score	13 (6 to 15)
**pH**		7.34 (7.29 to 7.39)
**SBE**	mmol/l	-2.0 (-4.0 to 0.0)
**Lactate**	mmol/l	1.7 (1.2 to 2.7)
**SatO**_**2 **_**(PH)**	%	98 (93 to 100)
**Shockindex (PH)**	HR/SBP	0.62 (0.50 to 0.75)
**Hemoglobin**	mmol/l	8.4 (7.3 to 9)
**Platelet count**	10^9^/l	208 (173 to 253)
**APTT > 35 sec**	%	8% (6)
**INR > 1.2**	%	13% (10)
**Saline (PH)**	ml	350 (0 to 1, 000)
**MT (> 10 RBC in 24 h)**	% (n)	14% (11)
**Mortality**	% (n)	18% (14)

Data are presented as medians (IQR) or n (%).

APTT, activated partial thromboplastin time; GCS, Glascow Coma Score scale; INR, international normalized ratio; ISS, injury severity score; MT, > 10 RBC the initial 24 hours; PH, pre-hospital at the site of injury; RBC, red blood cells; sTBI, severe Traumatic Brain Injury, Abbreviated Injury Score head > 3

### Trauma DIC

No patients had overt DIC according to the ISTH criteria [18,19] since only one patient had platelets between 50 and 100 *10^9^/l (one point), one patient had fibrinogen < 1 g/l (one point), no patients had D-dimer > 0.39 mg/l and four patients had INR between 1.4 and 2.3 (one point). No patients had more than one concurrent abnormal parameter so no patients scored more than one point. No attempts were made to stratify patients to compare these according to DIC criteria.

### Biomarker profile in patients with ACoTS

Twelve patients (15%) had ACoTS defined as APTT or INR above normal reference level [17,20,21] (Table 1). When comparing patients with or without ACoTS, those demonstrating ACoTS had a higher degree of tissue injury (higher ISS, hcDNA, Annexin V) and shock (lower pH and SBE), lower pre-hospital Glascow Coma Score (GCS), hemoglobin (*P *= 0.055), higher transfusion requirements and a four-fold increased mortality (Table 2). Patients with ACoTS also had higher sTM, syndecan-1, D-dimer and IL-6 but lower AT, PC, fibrinogen and FXIII levels indicative of enhanced endothelial cell and glycocalyx damage, factor consumption, hyperfibrinolysis and inflammation, respectively, and they had a tendency towards lower thrombin generation (PF1.2) and lower sEPCR and PS (Table 2).

**Table 2:** Demography, injury severity, transfusions, mortality, hemostasis and biomarkers of coagulopathy in ACoTS and non-ACoTS patients

		ACoTS	Normal (no ACoTS)	p to value
**Demography**				
**N**		12	68	
**Age**	yrs	42 (26 to 74)	46 (34 to 63)	NS
**Gender**	m% (n)	75% (9)	66% (45)	NS
**Blunt trauma**	% (n)	92% (11)	91% (62)	NS
**ISS**	score	34 (30 to 43)	17 (10 to 25)	**< 0.001**
**sTBI**	% (n)	25% (3)	32% (19)	NS
**GCS (PH)**	score	3 (3 to 7)	13 (7 to 15)	**< 0.001**
**Shockindex (PH)**	HR/SBP	0.68 (0.56 to 0.78)	0.61 (0.48 to 0.75)	NS
**RBC 1 h**	n	5 (0 to 9)	0 (0 to 0)	**< 0.001**
**MT (> 10 RBC in 24 h)**	% (n)	50% (6)	7% (5)	**< 0.001**
**Mortality**	% (n)	50% (6)	12% (8)	**0.001**
**Shock, biochemistry and coagulopathy**				
**pH**		7.27 (7.13 to 7.31)	7.36 (7.31 to 7.40)	**0.001**
**SBE**	mmol/l	-5.1 (-8.2 to -2.35)	-1.8 (-3.4 to 0.0)	**0.009**
**Hemoglobin**	mmol/l	6.8 (5.8 to 8.8)	8.5 (7.6 to 9.0)	0.055
**Platelet count**	10^9^/l	197 (173 to 238)	208 (176 to 259)	NS
**Fibrinogen**	g/l	1.53 (1.25 to 2.02)	2.45 (2.18 to 2.89)	**0.001**
**FXIII**	microg/ml	23 (17 to 29)	30 (24 to 39)	**0.004**
**vWF**	%	143 (100 to 209)	201 (140 to 226)	0.117
**APTT**	sec	33 (27 to 42)	25 (23 to 26)	NA
**INR**	ratio	1.3 (1.3 to 1.5)	1.1 (1.1 to 1.1)	NA
**Tissue, endothelial cell and glycocalyx injury**				
**Histone-complexed DNA**	%	15.4 (8.5 to 58.2)	4.8 (0.1 to 11.8)	**0.003**
**Annexin V**	ng/ml	45 (40 to 61)	24 (21 to 35)	**0.001**
**sTM**	ng/ml	2.90 (2.27 to 4.09)	1.43 (0.92 to 3.34)	**0.015**
**Syndecan-1**	ng/ml	62 (34 to 107)	31 (18 to 48)	**0.013**
**Thrombin generation**				
**PF1.2**	nmol/l	3.17 (0.61 to 16.03)	6.71 (2.08 to 18.79)	0.120
**TAT**	ng/ml	38 (36 to 41)	36 (30 to 43)	NS
**Natural anticoagulation**				
**AT**	10^3 ^U/l	0.68 (0.62 to 0.85)	0.95 (0.87 to 1.03)	**< 0.001**
**PC**	%	72 (60 to 89)	114 (99 to 129)	**< 0.001**
**APC**	ng/ml	9.98 (8.59 to 11.96)	9.78 (7.72 to 12.15)	NS
**sEPCR**	ng/ml	174 (141 to 242)	230 (175 to 398)	0.087
**PS**	%	57 (50 to 69)	66 (61 to 71)	0.078
**TFPI**	ng/ml	63 (43 to 74)	60 (47 to 80)	NS
**Fibrinolysis**				
**D-dimer**	ng/ml	174 (173 to 176)	158 (122 to 173)	**0.001**
**tPA**	ng/ml	7.2 (5.5 to 11.8)	6.9 (3.5 to 12.6)	NS
**PAI**	ng/ml	26 (11 to 37)	22 (14 to 40)	NS
**Inflamamtion**				
**sC5b-9**	ng/ml	1, 014 (701 to 1, 173)	1, 027 (905 to 1, 232)	NS
**IL-6**	pg/ml	110 (98 to 128)	61 (18 to 118)	**0.024**

Data are presented as medians (IQR) or n (%), with *P*-values shown for variables with *P *< 0.200, and in bold for *P *< 0.050. Patients with or without ACoTS were compared by Wilcoxon Rank Sum tests or Chi-square/Fischer exact tests, as appropriate.

APTT, activated partial thromboplastin time; GCS, Glascow Coma Score scale; INR, international normalized ratio; ISS, injury severity score; MT, > 10 RBC the initial 24 hours; RBC, red blood cells; sTBI, severe Traumatic Brain Injury, Abbreviated Injury Score head > 3; PH, pre-hospital at the site of injury.

Biomarker abbreviations, see Materials and methods section, ELISA and the List of abbreviations.

Though the proportion of patients with sTBI was comparable in ACoTS and non-ACoTS patients (Table 2), we investigated whether sTBI confounded the observed differences between ACoTS and non-ACoTS patients by excluding sTBI patients before the comparison, because it is widely debated whether sTBI may directly drive coagulopathy in trauma patients. When comparing the 9 ACoTS and 39 non-ACoTS patients without sTBI, these patients displayed the same differences in clinical presentation, outcome and biomarker profiles as those described for ACoTS and non-ACoTS patients in the full cohort. Thus, ACoTS/non-sTBI patients still had significantly higher ISS, transfusion requirements, mortality, enhanced shock (lower pH, SBE) and a biomarker profile indicative of enhanced tissue injury (higher hcDNA, Annexin V), factor consumption and bleeding (lower AT, PC, fibrinogen, FXIII, hemoglobin) and hyperfibrinolysis (D-dimer) (all *P *< 0.05) and thrombin generation remained borderline significantly reduced in ACoTS/non-sTBI patients (*P *= 0.125). In contrast to the finding in the full cohort, syndecan-1 and sTM were only borderline significantly increased in ACoTS/non-sTBI patients (*P *= 0.118 and *P *= 0.086), which is probably due to a reduction in power following exclusion of 31% of the patients rather than due to a biologically relevant difference.

### Injury severity, shock and biomarkers in ACoTS

Since trauma and shock are proposed drivers of ACoTS, we looked for differences in associations between ISS or SBE and the investigated biomarkers in patients with or without ACoTS.

With regards to ISS, Activated Protein C correlated positively with ISS in non-ACoTS (rho = 0.41, *P *= 0.001) but tended to correlate negatively in ACoTS (rho = -0.43, *P *= 0.161) patients. Likewise, PF1.2 tended to correlate positively with ISS in non-ACoTS (rho = 0.22, *P *= 0.076) but negatively in ACoTS (rho = -0.52, *P *= 0.086). Furthermore, only in non-ACoTS patients, higher ISS correlated positively with hcDNA, sTM, syndecan-1 (all *P *< 0.001), tPA (*P *= 0.026), D-dimer (*P *< 0.001) and IL-6 (*P *< 0.001) but negatively with AT (*P *= 0.018), fibrinogen (*P *= 0.009) and FXIII (*P *= 0.001) indicating that increasing injury severity in non-ACoTS patients was associated with a biomarker profile suggestive of some degree of TIC with protein C activation, coagulation factor consumption, hyperfibrinolysis and inflammation.

With regards to Standard Base Excess (SBE), Protein C and SBE correlated strongly positively in ACoTS (rho = 0.87, *P *< 0.001) whereas these only tended to correlate in non-ACoTS (rho = 0.21, *P *= 0.102). Activated Protein C did not correlate with SBE in any of the groups (data not shown). In non-ACoTS patients, SBE correlated negatively with Annexin V (*P *= 0.003), syndecan-1 (*P *= 0.004), D-dimer (*P *= 0.018) and IL-6 (*P *= 0.038) and positively with sEPCR (*P *= 0.029) (the latter also observed in ACoTS patients, *P *= 0.039).

### Fibrinogen and FXIII consumption

**Figure 1:**
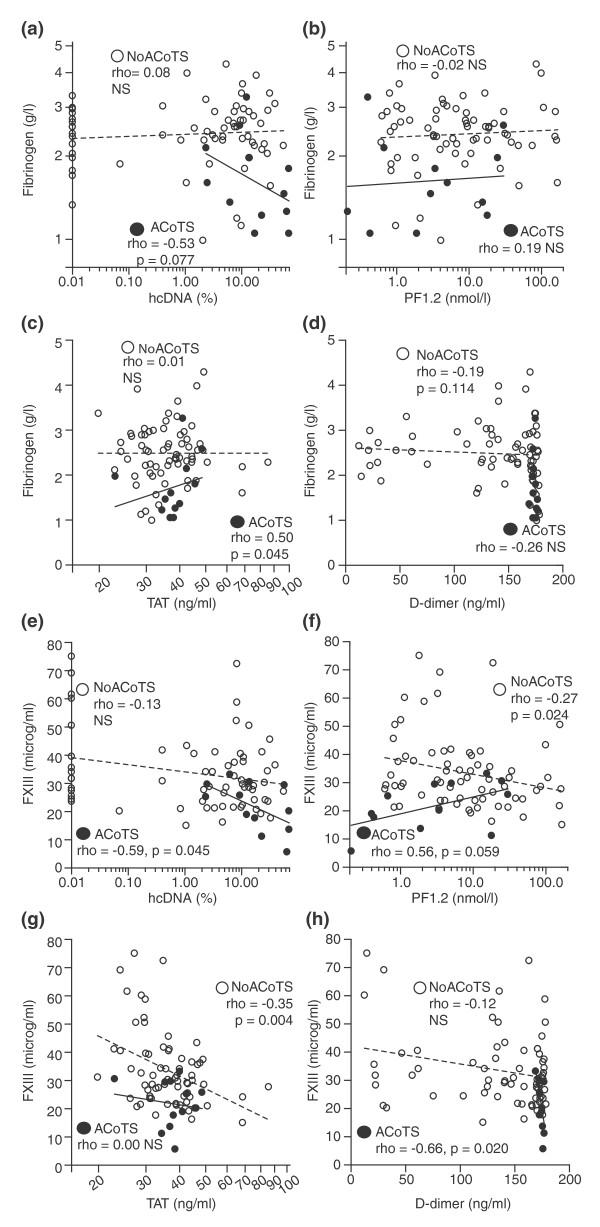
**Correlations between fibrinogen or FXIII and different biomarkers in ACoTS or non-ACoTS patients**. Correlations between fibrinogen **(A-D) **or FXIII **(E-H) **and biomarkers of tissue damage (hcDNA), thrombin generation (PF1.2, TAT) and fibrinolysis (D-dimer) on admission in 80 trauma patients stratified according to presence (ACoTS) or absence (no ACoTS) of ACoTS defined as APTT or INR above normal reference (*n *= 12). Rho and *P*-values are shown for correlations between fibrinogen or FXIII and the mentioned variables in patients with ACoTS (black circles, filled lines) or without ACoTS (white circles, dashed lines): A) log_10 _hcDNA (%) vs. fibrinogen (g/l), B) log_10 _PF1.2 (nmol/l) vs. fibrinogen (g/l), C) Log_10 _TAT (ng/ml) vs. fibrinogen (g/l), D) Log_10 _D-dimer (ng/ml) vs. fibrinogen (g/l), E) log_10 _hcDNA (%) vs. FXIII (microg/ml), F) log_10 _PF1.2 (nmol/l) vs. FXIII (microg/ml), G) Log_10 _TAT (ng/ml) vs. FXIII (microg/ml), H) Log_10 _D-dimer (ng/ml) vs. FXIII (microg/ml).

Given that ACoTS patients had low fibrinogen and FXIII levels, we looked for differences in associations between fibrinogen or FXIII and biomarkers of tissue damage, thrombin generation and fibrinolysis in patients with or without ACoTS. Notably, we found different correlations between fibrinogen/FXIII and hcDNA, PF1.2, Thrombin/antithrombin (TAT)-complexes and D-dimer in ACoTS and non-ACoTS patients (Figure 1A-H) revealing negative correlations between FXIII and hcDNA (Figure 1E) and D-dimer (Figure 1H) and positive correlations between fibrinogen and TAT (Figure 1C) only in ACoTS patients. Non-ACoTS patients displayed negative correlations between FXIII and biomarkers of thrombin generation (PF1.2, TAT, Figure 1F-G) indicating that different mechanisms may contribute to fibrinogen and FXIII consumption in patients with or without ACoTS.

## Discussion

In the present study, no patients had overt DIC according to ISTH criteria whereas 15% had ACoTS, and these patients had higher ISS, transfusion requirements and mortality, and a biomarker profile indicative of enhanced tissue, endothelial cell and glycocalyx damage, and consumptive coagulopathy with low AT, PC, fibrinogen and FXIII levels, hyperfibrinolysis and inflammation. Importantly, in non-ACoTS patients, higher ISS was associated with a biomarker profile indicative of enhanced tissue, endothelial cell and glycocalyx damage as well as an early hemostatic response/coagulopathy characterized by protein C activation, coagulation factor consumption, hyperfibrinolysis and inflammation suggesting that TIC represents a continuum of hemostatic response/coagulopathy dependent on the trauma hit and its downstream effects.

Recently, it has been intensively debated whether the early coagulopathy in severely injured patients reflects DIC with a fibrinolytic (hemorrhagic) phenotype [8], ACoTS [7,13] or yet other entities [9]. In the present study, we compared the prevalence of DIC and ACoTS in trauma patients admitted to our Level I Trauma Center directly from the scene of accident with admission blood samples taken approximately one hour post-injury and found that no patients had overt DIC according to ISTH criteria, whereas 15% had ACoTS. In accordance with some studies [11,22,23], but in contrast to others [15], patients with ACoTS had low fibrinogen and FXIII levels indicative of consumption coagulopathy and in ACoTS patients biomarkers of tissue damage (hcDNA), low thrombin generation (TAT) and hyperfibrinolysis (D-dimer) correlated with fibrinogen and FXIII consumption. The finding that hcDNA correlated negatively with coagulation factors in ACoTS patients is notable given the recent finding that extracellular nucleic acids can activate the contact activation (intrinsic) pathway directly since it indicates that high levels of extracellular nucleic acids derived from massive tissue and endothelial injuries may contribute significantly to coagulation factor consumption [24]. Furthermore, the finding that low SBE (shock) correlated strongly with low PC in ACoTS patients is in alignment with the notion that trauma-induced shock through hypoperfusion [15] and/or enhanced catecholamine surge [20] contributes to Protein C consumption probably through enhanced endothelial thrombomodulin expression and/or high circulating soluble thrombomodulin. The finding here of increased circulating syndecan-1, a biomarker of glycocalyx degradation [25], in ACoTS patients probably reflects more severe endothelial damage secondary to the trauma and its downstream effects that may augment consumption of both fibrinogen and FXIII by promoting a procoagulant endothelial surface and by enhancing the shedding of thrombomodulin [26]. Thereby, enhanced glycocalyx degradation could be a mechanistic difference between ACoTS defined by increased APTT/INR as compared to non-ACoTS. Thus, the coagulopathy in ACoTS may both reflect consumption of coagulation factors critical for clot generation [27-29] and endogenous anticoagulation driven by tissue injury, hyperfibrinolysis and/or shock (hypoperfusion, catecholamines) [17,20,26].

**Figure 2:**
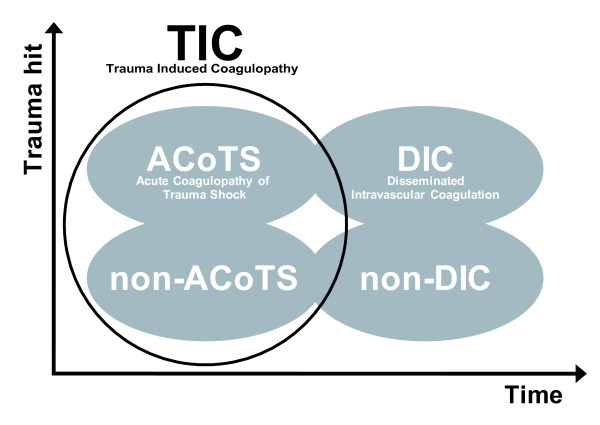
**Association between assessment time-point, trauma hit and type of coagulopathy in trauma patients**. Association between assessment time-point (blood sampling), trauma hit (tissue injury, shock) and trauma-induced coagulopathy (TIC), acute coagulopathy of trauma shock (ACoTS), non-ACoTS, disseminated intravascular coagulation (DIC) and non-DIC. We infer that TIC represents an early (minutes) progressive endogenous response to the trauma hit (tissue injury, shock) ranging from and covering both non-ACoTS and ACoTS (defined as moderately increased plasma based coagulation tests), with a biomarker profile indicative of endothelial and glycocalyx damage, factor consumption, hyperfibrinolysis and inflammation. The immediate hemostatic response to trauma is probably evolutionarily adapted to improve the chance of survival by, for example, inducing local hemostasis while preserving perfusion and oxygen delivery [16] with ACoTS representing an exaggerated non-adapted response in patients that without immediate life support would have succumbed. Progression to overt DIC does not appear to be part of the early response to trauma but may occur later (hours or days) post-injury, probably driven by a combination of the tissue injury exerted by the initial trauma hit, systemic endothelial dysfunction/damage, exhaustion of the natural anticoagulant pathways and/or excessive inflammation. Whether early TIC progresses to DIC may be determined by a combination of exogenous factors (the early trauma hit and later hits) [30] and endogenous factors (genetically determined response to the hits).

An important finding in the present study was that increasing ISS in patients without ACoTS (normal APTT/INR) was associated with a response resembling that described in patients with ACoTS [7,8,14,15,17,22,23], that is, enhanced tissue, endothelial cell and glycocalyx damage, protein C activation, factor and AT consumption, hyperfibrinolysis and inflammation. The lack of correlations between ISS and INR/APTT in non-ACoTS patients, however, emphasizes that the complexity of TIC and/or the hemostatic response to injury is not fully reflected by plasma based coagulation tests. The finding of comparable biomarker profiles in ACoTS and non-ACoTS patients with increasing injury severity suggests that these conditions may display a continuum of coagulopathy reflecting a progressive early evolutionarily adapted hemostatic response to the trauma hit with ACoTS probably representing an extreme response in patients with (from an evolutionary point of view) non-survivable injuries (Figure 2). Furthermore, we hypothesize that both ACoTS and non-ACoTS are part of TIC (Figure 2) which, based on the biomarker profile, includes endothelial cell and glycocalyx damage, PC activation, coagulation factor and AT consumption, hyperfibrinolysis and inflammation albeit this needs to be confirmed in an independent cohort of trauma patients of appropriate size. The discrepancy between studies reporting DIC vs. ACoTS may obviously reflect various methodological differences although we infer that the timing of the blood sampling post-injury is a critical factor since most studies reporting overt DIC in trauma patients have collected admission blood samples several hours (4 to 24 h) post-injury [11,12], whereas most studies reporting of ACoTS have collected admission blood samples much earlier (1 h or less post-injury) [14,15,22,23]. Given the dynamic nature of the hemostatic and inflammatory response to trauma [30], progression to overt DIC post-injury may occur hours (but not immediately) after the injury, probably driven by a combination of the tissue injury exerted by the initial trauma hit, systemic endothelial dysfunction/damage, exhaustion of the natural anticoagulant pathways and/or excessive inflammation (Figure 2).

The results presented here are subject to the limitations inherent to observational studies and, thereby, do not allow independent evaluation of cause-and-effect relationships. Furthermore, the low number of subjects and especially the low number of both severely injured patients and patients with ACoTS, included here increases the risk of introducing a type II error, emphasizing that the findings herein should be confirmed in a larger cohort of patients, and this is currently underway.

## Conclusions

The present study found that no trauma patients with blood sampled approximately one hour post-injury had overt DIC, whereas 15% had ACoTS defined as moderately increased APTT and/or INR. Though ACoTS patients had higher ISS, transfusion requirements and mortality and evidence of enhanced tissue, endothelial cell and glycocalyx damage, consumptive coagulopathy, hyperfibrinolysis and inflammation, non-ACoTS patients displayed a comparable biomarker profile with increasing injury severity. Based on these findings we infer that ACoTS and non-ACoTS represent a continuum of coagulopathy reflecting a progressive early evolutionarily adapted hemostatic response to the trauma hit and both are parts of TIC, whereas DIC does not appear to be part of this early response.

## Key messages

• It is debated whether severe tissue injury induces an immediate disseminated intravascular coagulation (DIC) or acute coagulopathy of trauma shock (ACoTS, increased APTT and/or INR).

• In 80 adult trauma patients investigated approximately one hour post injury, no patient had overt DIC (ISTH criteria), whereas 15% had ACoTS.

• ACoTS patients had higher ISS, transfusion requirements and mortality, and a biomarker profile suggestive of enhanced tissue, endothelial cell and glycocalyx damage and consumption coagulopathy with hyperfibrinolysis and inflammation, but non-ACoTS patients displayed the same profile with increasing ISS.

• Both ACoTS and non-ACoTS may represent a continuum of coagulopathy that reflects an evolutionarily adapted hemostatic response to the trauma hit.

• Both ACoTS and non-ACoTS are parts of trauma-induced coagulopathy (TIC) whereas DIC does not appear to be part of this early response.

## Abbreviations

ACoTS: acute coagulopathy of trauma shock; APC: activated Protein C; APTT: activated partial thromboplastin time; AT: antithrombin; DIC: disseminated intravascular coagulation; FDP: fibrin/fibrinogen degradation products; FV: coagulation factor V; FVIII: coagulation factor VIII; FXIII: coagulation factor XIII; GCS: Glasgow Coma Score; hcDNA: histone-complexed DNA fragments; ICU: intensive care unit; IL-6: interleukin-6; INR: international normalized ratio; IQR: interquartal range; ISS: Injury Severity Score; ISTH: International Society of Thrombosis and Haemostasis; Ly30: lysis 30 min after maximal amplitude is reached; MA: maximal amplitude; MECU: mobile emergency care units; PAI-1: plasminogen activator inhibitor-1; PC: Protein C; PF1.2: prothrombinfragment 1 and 2; PS: Protein S; PT: prothrombin time; RBC: red blood cells; RT: room temperature; RTS: revised trauma score; SBE: standard base excess; sC5b-9: terminal complement complex; sEPCR: soluble endothelial protein C receptor; sTBI: severe traumatic brain injury; sTM: soluble thrombomodulin; sTAT-complex: thrombin/antithrombin complex; TARN: Trauma Audit & Research Network; TEG: thrombelastography; TFPI: tissue factor pathway inhibitor; TIC: trauma-induced coagulopathy; tPA: tissue-type plasminogen activator; vWF: von Willebrand factor

## Competing interests

The authors declare that they have no competing interests.

## Authors' contributions

PIJ contributed to the conception and design of the study, interpretation of data and writing of the manuscript. AMS, AP, KLW, MW and CFL contributed to the design of the study and critically revised the manuscript. SRO contributed to the design of the study, statistical data analyses, interpretation of data, figure drafting and writing of the manuscript. All authors read and approved the final manuscript.
